# Structural equation model of the effect of biological maturation on metabolic syndrome risk and C-reactive protein: effect of trunk fat and sports participation

**DOI:** 10.1038/s41598-021-97034-8

**Published:** 2021-09-10

**Authors:** André O. Werneck, Enio R. V. Ronque, Rômulo A. Fernandes

**Affiliations:** 1 Laboratory of InVestigation in Exercise – LIVE, Department of Physical Education, Universidade Estadual Paulista “Júlio de Mesquita Filho” (UNESP), Rua Roberto Símonsen, 305, Presidente Prudente, 19060-900 Brazil; 2 Physical Activity and Health Laboratory, Department of Physical Education, Londrina State University, Londrina, Brazil

**Keywords:** Endocrine system and metabolic diseases, Cardiology

## Abstract

Our aim was to analyze the association between somatic maturation and alterations in metabolic syndrome (METs) risk and C-reactive protein (CRP), focusing on the effect of changes in trunk fat and sports practice. This was a longitudinal study with a one-year follow-up. The sample was composed of 139 adolescents (46 without sports participation and 93 young athletes), aged 10–17 years. As outcomes, we adopted CRP and METs risk (triglycerides, HDL-c, fasting glucose, and mean blood pressure). Somatic maturation was estimated using Mirwald’s method. Structural equation models were used. Somatic maturation was not associated with sports practice, trunk fat, METs risk neither CRP. Sports practice was associated with a reduction in METs risk (β = −0.926; 95%CI:−1.773, −0.080) and reduction in trunk fat (−10.957; −19.630, −2.283), which was associated with increases in METs risk (0.020; 0.004, 0.036). In the CRP model, sports practice was associated with a reduction in trunk fat (−10.324; −18.637, −2.010), which in turn was associated with a reduction in CRP (0.007; 0.001, 0.013). Sports practice and lower trunk adiposity were associated with reductions in trunk fat, METs risk, and CRP through direct and indirect pathways. Our findings highlight the role of sports practice in attenuating the negative effect of trunk adiposity.

## Introduction

The components of metabolic syndrome (aggregation of three or more components: central obesity, high blood pressure, insulin resistance, and dyslipidemia) and elevated inflammation markers are increasingly prevalent worldwide, even among pediatric groups^1–3^.

Especially during adolescence, due to its impact on all biological tissues and organs of the human body towards the mature state, the biological maturation process is a major contributor to the adoption of relevant behaviors, such as sports participation^4^ (most common manifestation of physical exercise during adolescence), but also a possible risk factor for the onset of chronic diseases. In this sense, previous findings have already shown that adolescents with earlier maturation have higher cardiovascular risk factors during adolescence, as well as prospectively during adulthood^5–9^, which can be caused especially through the increase in adiposity and the adoption of risk behaviors^10,11^. Therefore, identifying early life risk factors for the development of chronic diseases is highlighted for prevention strategies.

Childhood obesity is a factor highlighted for its association with several cardiovascular risk factors, including metabolic syndrome^2,12^. On the other hand, higher physical activity level is a protective factor for metabolic syndrome components among adolescents^13^, mainly when considering practice in moderate-to-vigorous intensity domains, such as observed during sports participation^14^, which acts both through direct pathways and through indirect mechanisms such as energetic regulation^15^. In this sense, sports participation is also encouraged during adolescence as a cardiovascular protective factor for diseases in adulthood through methylation pathways and also by dependent pathways, such as through the maintenance of physical activity and control of adiposity^16–18^.

Considered in an integral manner, the prediction of both metabolic and inflammatory risk factors can be determined by several biological and behavioral factors during growth, taking into account the relationship of biological maturation with physical activity level and adiposity^19^. Previous findings suggested that adiposity is a clear mediator of the association between biological maturation and risk of metabolic syndrome^11,20^. However, the majority of previous studies assessed this phenomenon using cross-sectional approaches, reducing the cause-effect associations^9,11,20^. Moreover, the role of sports participation in this complex association is still unclear.

Thus, our aim is to analyze the association between somatic maturation and alterations in metabolic syndrome risk and C-reactive protein (CRP) among adolescents, focusing on the effect of changes in trunk adiposity and sports participation.

## Materials and Methods

### Sampling

In 2017, 285 adolescents were recruited from four school units and nine sport clubs. For the selection process, those responsible for the selected school units and sports facilities were contacted and informed about the implementation of the research project, as well as the inclusion criteria. After receiving permission, the adolescents were invited to participate. The following inclusion criteria were adopted in the present study: (i) Being aged between 10 and 17 years; (ii) Not presenting any clinical or metabolic disorder (previously diagnosed) that may influence the practice of habitual physical activity; (iii) The legal guardian signed the consent form.

From the 192 adolescents who completed both waves (baseline and 2018 follow-up), 22 adolescents failed to complete the inclusion criteria of the present study and 31 other adolescents failed to provide complete data on metabolic syndrome components and CRP. Therefore, 46 controls, 5 track and field athletes, 7 baseball athletes, 14 basketball athletes, 9 artistic gymnastics athletes, 3 judo athletes, 12 karate athletes, 11 kung-fu athletes, 19 swimming athletes, and 13 tennis players composed the final sample. The study was approved by the local Ethics Committee of research involving humans, in accordance with the principles of the Declaration of Helsinki. Informed consent was obtained from all individual participants and included in the study as well as from a parent or legal guardian.

### Metabolic syndrome risk and CRP

Blood samples were collected during the morning period, following a 12-h fasting period, by trained professionals from a private laboratory, which has all the certifications requested by the Brazilian Ministry of Health. Blood analyses followed the recommendations of the III Brazilian Guideline on Dyslipidemias and the Atherosclerosis Prevention Guideline of the Atherosclerosis Department of the Brazilian Society of Cardiology. The following components were evaluated: high-density lipoprotein (HDL-c), triglycerides (TG), and fasting glucose and insulin (fasting glucose and insulin were used to calculate the Homeostatic Model Assessment of Insulin Resistance [HOMA-IR]). Blood pressure was assessed using an automatic right arm after a rest period of 10 min using a validated automatic equipment (OMRON, HEM-742)^21^. Three measurements were performed at 2-min intervals. Blood pressure was determined based on the arithmetic mean of the two last measurements. To describe the characteristics of the sample, we estimated the prevalence of metabolic syndrome, using the International Diabetes Federation classification^22^.

Plasma CRP concentration was assessed by the immunoturbidimetric method using an enzymatic kit (Millipore, St Charles, MO [inter and intra-assay coefficients ranging from 4.6 to 6.0%, respectively]). For the main analyzes, CRP and metabolic syndrome risk score were considered as outcomes, which was calculated by summing the z-scores of the metabolic syndrome variables (inverted HDL-C, triglycerides, HOMA-IR, mean arterial pressure [mean z-score of systolic and diastolic blood pressure])^23^. Having in mind that it is not expected that adolescents present metabolic syndrome itself, the metabolic syndrome score was proposed^23^, which indicates if the participant has a higher or lower metabolic syndrome risk, considering the whole group^23^. Abdominal adiposity was removed from the metabolic syndrome risk score, as it is plausible that including abdominal adiposity in the outcome would overestimate the effect of abdominal adiposity as a exposure^24^. For both outcomes, the difference between baseline and follow-up was used in the models.

### Somatic maturation

We estimated somatic maturation as an indicator of biological maturation, using the age of peak height velocity (PHV). The estimation of PHV uses four anthropometric variables (stature, sitting height, leg length, and body mass)^25^. These equations provide the time (in years) that is missing (negative values) or past (positive values) from PHV (the moment of highest height gain during adolescence), which is characterized as an important biological event present in the process of human maturation. In this sense, by subtracting the PHV from the chronological age, the age of PHV is obtained^25^.

### Trunk fat

Trunk fat was assessed using dual-energy x-ray absorptiometry (Lunar DPX-NT; General Electric Healthcare, Little Chalfont, Buckinghamshire, UK) with GE Medical System Lunar software (version 4.7). The measure considered the trunk area and the amount of fat, in percentage (%), in this body segment. The scanner quality was tested by a trained researcher before each day of measurement, following the manufacturer's recommendations and all scans were carried out at the university laboratory in a temperature-controlled room. The participants wore light clothing, without shoes, and remained in the supine position on the machine (approximately 15 min). The measurement was conducted by a single previously trained evaluator (same evaluator as the initial 2017 collections). The precision of the machine in terms of coefficient of variation was 0.66% (n = 30 subjects not involved in this study).

### Covariates

Covariate factors for this study were sex (boy or girl), stature, chronological age (years), maternal schooling, body mass index, sugary foods consumption and transport physical activity. Stature was assessed using a digital scale (Professional model, Sanny®, Brazil, accurate to 0.1 cm). Maternal schooling (highest academic achievement) was reported by the adolescents and classified as up to high school or higher than high school. Body mass index was estimated using values of body mass and stature (body mass—in kg / stature^2^—in meters). Sugary foods consumption was assessed though questions about the frequency of biscuits, cake, candies, pasta, soft drinks and artificial juices consumption during the last seven days, using a 3-items Likert scale (1- did not eat, 2- sometimes or 3- almost every day). The score of each food consumption item were summed and a final score was created, ranging between six (lowest consumption) and 18 (highest consumption). Leisure and transport physical activities were estimated using the Baecke questionnaire^26^, the questionnaire contains questions regarding occupational, sports and exercise as well as leisure and transport activities. Considering the characteristics of the present study, which included participants practicing sports or not practicing exercise or sports, we adopted only the leisure and transport domain as covariate (4 items about time spent watching television; and time spent walking and cycling for commuting), which are combined in the same domain score. All the covariates were assessed during the baseline. Statistical models were simultaneously adjusted by all these factors.

### Statistical procedure

Values of mean, frequency, and standard deviation were used for sample description. Mann–Whitney and chi-square tests were used to compare groups. Partial correlation analyses were used to analyze the correlation between exposures, mediators, and outcomes.

**Figure 1:**
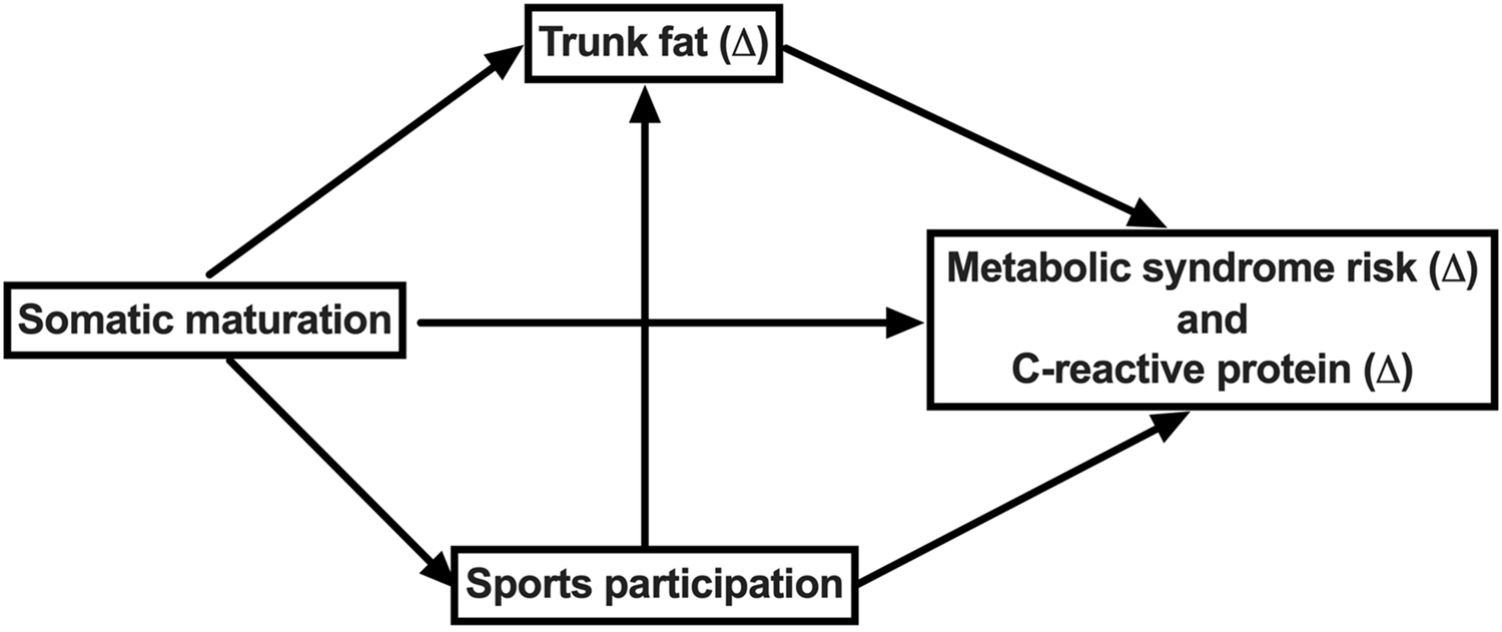
Theoretical model. Δ represents the change from baseline to follow-up.

The independent and mediation effects of the association between biological maturation, adiposity, and physical activity in the prediction of metabolic syndrome score were assessed using the maximum likelihood method. To evaluate the model fit, the parameters of the chi-square test, mean square root approximation, comparative fit index, and Tucker-Lewis index were analyzed according to recommendations^27^. All analyses were adjusted for sex, chronological age, and stature. Possible mediating effects were analyzed through resampling tests using the bootstrap method with 1,000 resamples. The structural equation model (theoretical model) of the association between age of PHV and alterations (Δ) in metabolic syndrome risk, considering sports participation and changes in trunk fat as potential mediators, is presented in Fig. 1. All analyzes were estimated using the software Stata 15.1.

### Ethical standards

The study was approved by the Ethics Committee of research involving humans from the Universidade Estadual Paulista “Júlio de Mesquita Filho” (Process: 1.677.938/2016), in accordance with the principles of the Declaration of Helsinki.

### Informed consent

Informed consent was obtained from all individual participants and included in the study as well as from a parent or legal guardian.

## Results

Considering missing data as well as outliers, the final sample was composed of 139 adolescents with complete data for metabolic syndrome risk and CRP analysis. Characteristics of the sample are presented in Table 1. Girls presented a lower age of PHV, fasting glucose, systolic blood pressure, and CRP, as well as higher trunk fat than boys. We did not find any case of metabolic syndrome.

**Table 1:** Characteristics of the boys and girls.

Variables	Boys (n = 88)	Girls (n = 51)	p
**Baseline**			
General			
Chronological age, y	14.5 ± 2.0	14.7 ± 2.0	0.755
Stature, cm	168.8 ± 10.8	160.1 ± 8.4	< 0.001
Body mass, kg	58.5 ± 12.7	52.0 ± 10.7	0.002
Body mass index, kg/m^2^	21.2 ± 4.0	20.6 ± 3.1	0.375
Maternal schooling—more than high school, %	37.5%	33.3%	0.622
Sugary foods consumption, score	12.0 ± 2.1	11.3 ± 1.9	0.097
Age of PHV, y	13.781.0	12.7 ± 1.0	< 0.001
Sports participation—yes (%)	65.9%	68.6%	0.743
Transport physical activity, score	2.5 ± 0.7	2.3 ± 0.7	0.023
Trunk fat, %	20.6 ± 10.0	31.4 ± 10.3	< 0.001
Metabolic			
HDL-C, mg/dL	53.9 ± 12.4	53.9 ± 10.1	0.530
Triglycerides, mg/dL	68.9 ± 21.7	74.2 ± 25.4	0.318
Fasting glucose, mg/dL	85.6 ± 7.7	81.4 ± 6.5	0.002
Inflammatory and cardiovascular			
Systolic blood pressure, mmHg	114.1 ± 12.1	106.5 ± 8.6	< 0.001
Diastolic blood pressure, mmHg	61.8 ± 7.0	62.5 ± 6.7	0.469
CRP, mg/dL	3.0 ± 2.1	2.0 ± 1.4	0.003
**Follow-up**			
General			
Stature, cm	172.3 ± 9.12	161.6 ± 7.0	< 0.001
Body mass, kg	63.0 ± 12.6	55.9 ± 11.2	< 0.001
Body mass index, kg/m^2^	21.1 ± 3.6	21.3 ± 3.5	0.813
Trunk fat, %	18.8 ± 9.2	31.6 ± 9.8	< 0.001
Metabolic			
HDL-C, mg/dL	53.1 ± 10.5	53.0 ± 11.1	0.896
Triglycerides, mg/dL	70.4 ± 26.6	71.0 ± 29.0	0.875
Fasting glucose, mg/dL	85.0 ± 6.4	82.1 ± 6.4	0.004
Inflammatory and cardiovascular			
Systolic blood pressure, mmHg	118.4 ± 12.3	106.8 ± 9.6	< 0.001
Diastolic blood pressure, mmHg	64.5 ± 7.3	62.4 ± 6.9	0.104
CRP, mg/dL	3.1 ± 2.1	3.3 ± 2.0	0.650

Values are presented using means, frequencies, and standard deviations. PHV, peak of height velocity. CRP, C-reactive protein.

Partial correlations (adjusted for sex, stature, and chronological age) are presented in Table 2. Sports participation was correlated with lower trunk fat (r = −0.204; p = 0.019) and lower risk of metabolic syndrome (r = −0.218; p = 0.012). In addition, higher trunk fat was correlated with higher risk of metabolic syndrome (r = 0.240; p = 0.006).

**Table 2:** Partial correlation (adjusted for sex, stature, chronological age, maternal schooling, body mass index, sugary foods consumption and transport physical activity) describing the relationship between exposure, mediators, and outcomes.

	1	2	3	4	5
1. Age of PHV	–	–	–	–	–
2. Sports participation *p*-value	*r* = −0.040 0.646	–	–	–	–
3. Trunk fat change *p*-value	*r* = −0.025 0.778	***r* = **−**0.204** **0.019**	–	–	–
4. METs risk change *p*-value	*r* = −0.020 0.821	***r* = **−**0.218** **0.012**	***r* = 0.240** **0.006**	–	–
5. CRP change *p*-value	*r* = −0.056 0.523	*r* = 0.147 0.092	*r* = 0.175 0.044	*r* = −0.069 0.434	–

Values are presented using correlation coefficients. PHV, peak of height velocity. METs, metabolic syndrome. CRP, C-reactive protein. Bold values represent *p* < 0.05.

**Figure 2:**
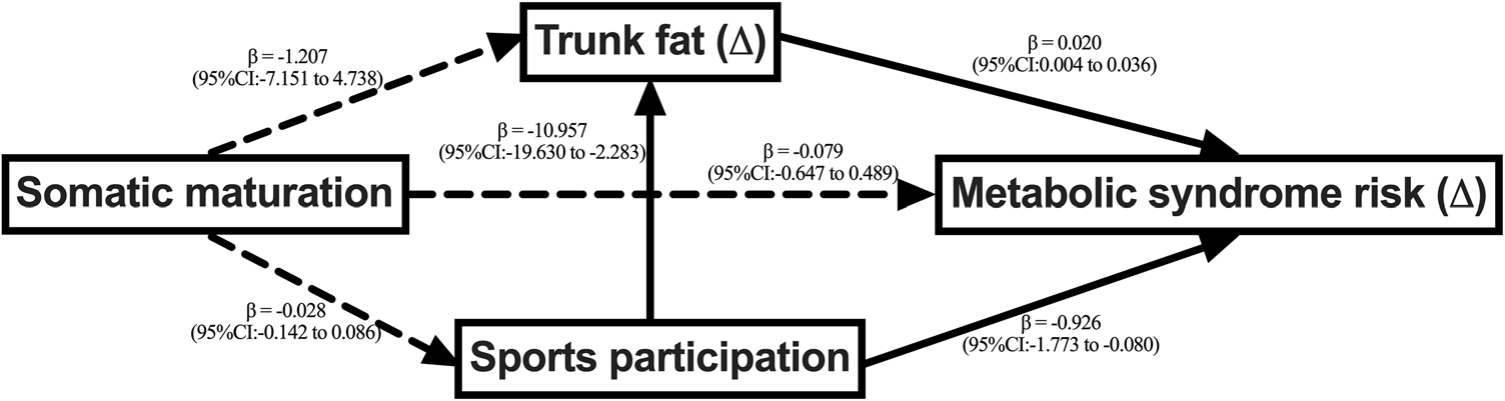
Structural equation model of the association between somatic maturation and metabolic syndrome risk considering trunk fat and sports practice as mediators. Note. Model adjusted for sex, stature, chronological age, maternal schooling, body mass index, sugary foods consumption and transport physical activity. Root mean square error of approximation: < 0.001, comparative fit index: 0.99, Tucker-Lewis index: 0.99.

Age of PHV was not associated with sports practice, trunk fat, and metabolic syndrome risk (Fig. 2). On the other hand, sports practice was associated with a reduction in metabolic syndrome risk (β = −0.926; 95%CI = −1.773 to −0.080), as well as with a reduction in trunk fat (β = −10.957; 95%CI = −19.630 to −2.283), which was associated with increases in metabolic syndrome risk (β = 0.020; 95%CI = 0.004 to 0.036).

**Figure 3:**
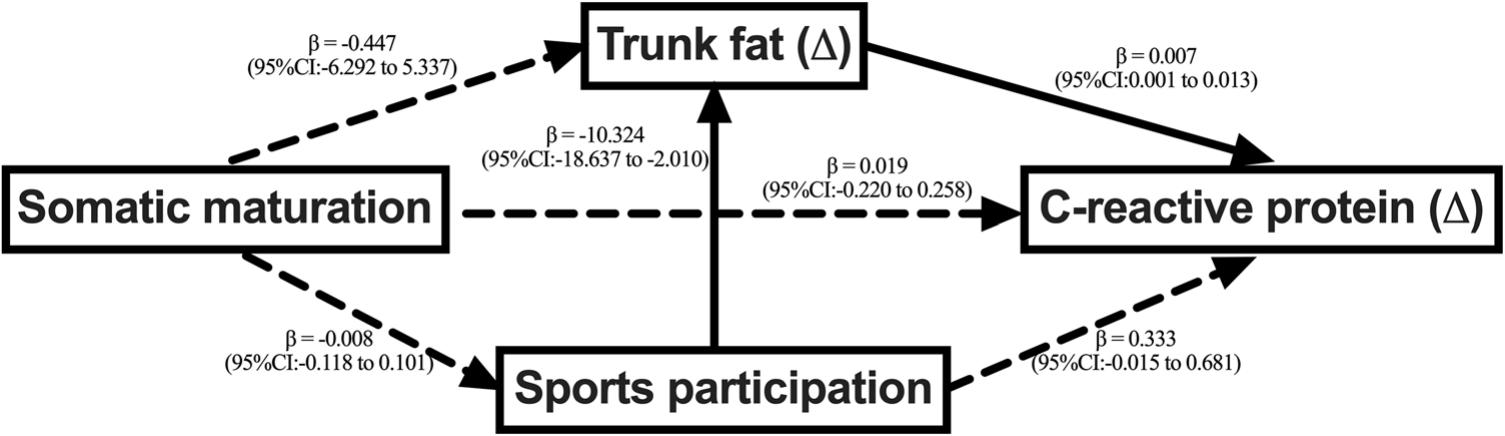
Structural equation model of the association between somatic maturation and c-reactive protein considering trunk fat and sports practice as mediators. Note. Model adjusted for sex, stature, chronological age, maternal schooling, body mass index, sugary foods consumption and transport physical activity. Root mean square error of approximation: < 0.001, comparative fit index: 0.99, Tucker-Lewis index: 0.99.

Figure 3 presents the structural equation model of the association between age of PHV and alterations in CRP over time, considering sports practice and changes in trunk fat as potential mediators. Age of PHV was not associated with sports practice, trunk fat, and CRP. However, sports practice was associated with reductions in trunk fat (β = −10.324; 95%CI = −18.637 to −2.010), which in turn was associated with a reduction in CRP (β = 0.007; 95%CI = 0.001 to 0.013).

## Discussion

The present study aimed to analyze the association between somatic maturation and prospective changes in metabolic syndrome risk and CRP, considering sports participation and trunk fat as potential mediators through a structural equation model. In this sense, our main findings were: (i) somatic maturation was not associated with the outcomes or mediators; (ii) increased trunk fat over time was directly associated with both increased metabolic syndrome risk and increased CRP; (iii) sports participation presented direct and indirect associations with changes in the risk of metabolic syndrome and CRP.

Biological maturation is a process marked by several alterations in all tissues of the human body^4^. In addition, the timing of biological maturation is associated with several health outcomes, such as higher body fat among early maturing adolescents^28^. Adolescents with earlier biological maturation also present cardiovascular risk factors such as higher metabolic syndrome risk through direct and indirect roles, given that adiposity, especially in the trunk region, is a recognized mediator^6,9,11,20^. Early biological maturation is also associated with the adoption of unhealthy behaviors, such as alcohol intake, tobacco use, and lower physical activity^10,19^. As sports participation is an important domain of physical activity among young people^29^, mainly because it is usually performed at moderate or vigorous intensity, it may be a possible mediator of the association between biological maturation and health risk outcomes. However, we found that, unlike previous studies, somatic maturation was not associated with any of the mediators or outcomes.

Our study highlights the role of sports participation during adolescence as an important protective factor for both metabolic syndrome risk and CRP through direct and indirect roles. There may be several mechanisms underlying the association of sports practice with alterations in metabolic syndrome risk and inflammation. Firstly, the reduction in body fat, through the regulation of energy expenditure, may be an important role, as observed in the present study^15^. In addition, sports practice is associated with reductions in inflammatory levels^30^, through the reduction in some cytokines such as IL-6 and adiponectin^31^, which are, in turn, associated with metabolic syndrome risk^32^. Moreover, intensity seems a key point to determine how effectively sports participation can impact metabolic aspects in adolescents, in which sports performed at higher physical demand tend to be more beneficial to different lipid and glycemic components than those performed at lower demand, including HDL-c^33^.

Trunk adiposity was characterized as the main theoretical mediation pathway of the association between somatic maturation and risk of metabolic syndrome, as well as which CRP^11,20^ was also a mediator of the association between sports participation and metabolic syndrome risk and CRP. Both outcomes may present convergent pathways, with higher body fat being associated with the release of pro-inflammatory cytokines^34^, elevating CRP levels as well as being associated with metabolic syndrome risk.

The lack of association of somatic maturation with sports participation, adiposity, and metabolic syndrome risk found in previous studies could be due to the selection bias of sports practice. In this sense, biological maturation has an effect on selection process as well as success in youth sports practice, in which taller and stronger youth athletes tend to be selected and obtain success^35,36^. Therefore, as we included youth athletes in our sample, it is expected that there was already a maturation bias, including lower adiposity variability among sports practitioners.

The current study has clear practical implications, such as highlighting sports participation as an important protective factor for metabolic syndrome risk, trunk fat, and CRP. In this sense, sports engagement during childhood and adolescence should be encouraged for cardiovascular risk prevention.

Our findings should be interpreted in light of potential limitations. Despite the advance of causality with a one-year follow-up, the present study presents only two waves, which may be a potential limitation for causality inference. In addition, somatic maturation is only one indicator of biological maturation^4^ and has potential sources of bias among adolescents with tall or short stature and adolescents who are distant from the PHV^37,38^. Also, the reduced sample limited the separation into subgroups, such as by age group and sex. Finally, there are unmeasured/unobserved confounders, which were not taken into account in the models, such as nutritional factors, socioeconomic status, and total physical activity, among others. On the other hand, we presented one year of follow-up with blood measurements, including an indicator of inflammation in Brazilian adolescents, which we consider to be a strong aspect of the study.

Thus, sports participation was associated with reductions in the risk of metabolic syndrome, trunk fat, and CRP through direct and indirect pathways. In addition, changes in trunk fat over time were directly associated with alterations in metabolic syndrome risk and CRP. On the other hand, somatic maturation was not associated with sports practice, changes in trunk fat, metabolic syndrome risk, and CRP. Future studies should adopt longer follow-up measures, with a higher number of waves, as well as including other risk behaviors in the model.
